# Neuroprotective Effects of Early Hypothermia Induced by Phenothiazines and DHC in Ischemic Stroke

**DOI:** 10.1155/2021/1207092

**Published:** 2021-01-18

**Authors:** Yun Han, Xiao-kun Geng, Hangil Lee, Fengwu Li, Yuchuan Ding

**Affiliations:** ^1^Luhe Institute of Neuroscience, Capital Medical University, Beijing, China; ^2^Department of Neurology, Luhe Clinical Institute, Capital Medical University, Beijing, China; ^3^Department of Neurosurgery, Wayne State University School of Medicine, Detroit, Michigan, USA; ^4^Department of Research & Development Center, John D. Dingell VA Medical Center, Detroit, Michigan, USA

## Abstract

**Methods:**

Adult male Sprague Dawley rats were studied in 4 groups: (1) sham; (2) stroke; (3) stroke treated with pharmacological hypothermia before reperfusion (interischemia hypothermia); and (4) stroke treated with pharmacological hypothermia after reperfusion is initiated (inter-reperfusion hypothermia). The combination of chlorpromazine and promethazine with dihydrocapsaicin (DHC) was used to induce hypothermia. To compare the neuroprotective effects of drug-induced hypothermia between the interischemia and inter-reperfusion groups, brain damage was evaluated using infarct volume and neurological deficits at 24 h reperfusion. In addition, mRNA expressions of NADPH oxidase (NOX) subunits (gp91^phox^, p67^phox^, p47^phox^, and p22^phox^) and glucose transporter subtypes (GLUT1 and GLUT3) were determined by real-time PCR at 6 and 24 h reperfusion. ROS production was measured by flow cytometry assay at the same time points.

**Results:**

In both hypothermia groups, the cerebral infarct volumes and neurological deficits were reduced in the ischemic rats. At 6 and 24 h reperfusion, ROS production and the expressions of NOX subunits and glucose transporter subtypes were also significantly reduced in both hypothermia groups as compared to the ischemic group. While there were no statistically significant differences between the two hypothermia groups at 6 h reperfusion, brain damage was significantly further decreased by interischemia hypothermia at 24 h.

**Conclusion:**

Both interischemia and inter-reperfusion pharmacological hypothermia treatments play a role in neuroprotection after stroke. Interischemia hypothermia treatment may be better able to induce stronger neuroprotection after ischemic stroke. This study provides a new avenue and reference for stronger neuroprotective hypothermia before vascular recanalization in stroke patients.

## 1. Introduction

Stroke is a leading cause of severe disability and a serious threat to human health worldwide. It has caused serious social and economic burdens [1, 2]. Super-early (4.5 hours) rt-PA intravenous thrombolysis is currently the most commonly used method of treatment for acute cerebral infarction. However, because of the narrow “time window,” high bleeding risk, inadequate national health consciousness, poor medical resource distribution, and problematic traffic congestion in our country, the actual thrombolysis rate is less than 30% [3]. Because of these limitations, a majority of patients cannot get timely and effective treatment [4]. Intra-arterial contact thrombolysis, mechanical thrombolysis, direct stent implantation, and other technologies have gradually appeared in recent years, hence extending the treatment time window and enabling the recanalization rate of occluded vessels to reach 71% [5–7]. However, the favorable prognosis in patients with recanalization was still less than 46%.

The core principle of acute ischemic cerebral infarction treatment is twofold: to prevent the expansion of irreversible injury and to save reversible ischemic tissue (also known as ischemic penumbra) [8]. Hypothermia therapy is currently recognized as one of the few effective neuroprotective strategies in the world [9–14]. In fact, due to its positive neuroprotective effects, mild hypothermia has been used worldwide for brain protection after neonatal hypoxic ischemic encephalopathy [15, 16], traumatic brain injury [17, 18], and cardiac resuscitation [19]. Clinically, the major limitations of physical hypothermia are the delays in cooling initiation and onset of target temperature. The late start necessitates prolonged cooling duration, which requires extensive medical and nursing efforts, and causes secondary complications [20]. Considering interischemia hypothermia, its benefits have been shown in cardiac studies, where it has improved the prognosis of patients [21]. However, it is not known whether interischemia hypothermia reduces tissue damage in acute ischemic stroke.

Studies have shown that therapeutic hypothermia can play a neuroprotective role by inhibiting the activity of nicotinamide adenine dinucleotide phosphate (NADPH) oxidase (NOX) and reducing the generation of reactive oxygen species (ROS) [22, 23]. NOX activation is an important mechanism of brain injury, which can be further aggravated by high glucose metabolism after stroke. After the occurrence of ischemic stroke, ATP in the penumbra is rapidly exhausted, and the brain tissue produces a large amount of reduced NADPH through the anoxic metabolic pathway through the hexose phosphate bypass. Under the action of NOX, a large amount of ROS is produced, leading to oxidative stress injury [24–26]. Previous studies have proved that NOX gene knockout mice [27] or NOX inhibitor intervention [28] can successfully inhibit ROS production and thereby reduce the oxidative stress injury after ischemia [29, 30]. In addition, our previous studies have found that drug-induced hypothermia after reperfusion inhibited the activity of NOX, reduced the production of ROS, and reduced the consequent oxidative stress, so as to play a neuroprotective role [31].

In this study, we determined whether interischemia hypothermia induced by the pharmacological approach induced stronger neuroprotection in the brain through inhibiting the activity of NOX and reducing the production of ROS. If successful, this study on the treatment of acute cerebral infarction before recanalization provides a reference for effective treatments and provides a basis for clinical transformation. In this way, stroke patients could receive drug hypothermia treatment with less medical and nursing efforts before vascular recanalization to protect the brain from further injury.

## 2. Materials and Methods

### 2.1. Subjects

**Figure 1:**
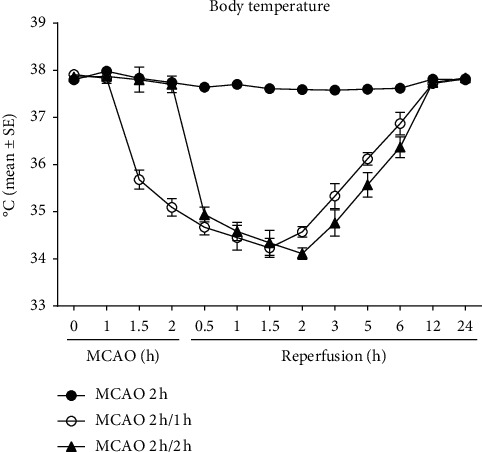
Body temperatures of 2 h MCAO rats with or without hypothermia at different time points.

All experimental protocols were approved by the Animal Care and Use Committee, Capital Medical University, Beijing, China, according to the National Institutes of Health (NIH, Bethesda, MD, USA) Guide for the Care and Use of Laboratory Animals. All adult male Sprague Dawley rats (280–320 g, Vital River Laboratory Animal Technology Co., Ltd., Beijing, China) were randomly divided into 4 groups (Figure 1(a)): (1) sham-operated group without middle cerebral artery occlusion (MCAO); (2) stroke group without pharmacological hypothermia (MCAO 2 h); (3) stroke group treated with pharmacological hypothermia 1 h before reperfusion (interischemia hypothermia) (MCAO 2 h/1 h); and (4) stroke group treated with pharmacological hypothermia after reperfusion is initiated (inter-reperfusion hypothermia) (MCAO 2 h/2 h). Each group was further divided into three subgroups, with 8 rats in each subgroup. The combination of chlorpromazine and promethazine with dihydrocapsaicin (DHC) was used to induce hypothermia. All experimental procedures and data analysis were performed in a randomized and blinded manner. At 24 h reperfusion, neurological deficits and infarct volume were examined in each group, and biochemical assays were performed.

### 2.2. Focal Cerebral Ischemia

The procedures have been described previously by us [31]. The animals underwent fasting for 12 h before the operation began. Animals were anesthetized in a chamber with 1–3% isoflurane along with a mixture of 70% nitrous oxide and 30% oxygen and maintained with 1% isoflurane. A 2 h right MCAO was induced using an intraluminal filament [32]. During operation, body temperature (rectal temperature), blood pH, pCO_2_ and pO_2_, and mean arterial pressure (MAP) were all monitored. Laser Doppler was used to monitor blood flow in the MCA-supplied region to ensure the success of the model.

### 2.3. Pharmacological Hypothermia

In all ischemia models with 2 h MCAO following reperfusion, a 1 : 1 ratio of chlorpromazine and promethazine (C + P) at 4 mg/kg in 3 ml of saline combined with dihydrocapsaicin (DHC) at doses of 0.5 mg/kg [33] was injected intraperitoneally, as described by us previously [31] at 1 or 2 h after the onset of ischemia. In order to maintain and enhance the efficacy of the drugs, a second injection with 1/3 of the initial dose was delivered in 2 h.

### 2.4. Body Temperature Monitoring

Rectal temperature (body temperature) was monitored in 30-minute or 1 h increments from before hypothermia until it returned to the initial levels.

### 2.5. Cerebral Infarct Volume

The rats were anesthetized with 1% chloral hydrate, and the brain tissue of the ischemic rats was removed and immediately sliced into seven coronal sections (2 mm thick), as described previously by us [34]. The sections were stained with 2,3,5-triphenyltetrazolium chloride (TTC, Sigma, USA) at 37°C. ImageJ image analysis software was used to measure cerebral infarction volume. In order to reduce the error caused by cerebral edema, the cerebral infarction volume was measured indirectly using the indirect calculation method, relative to the noninfarcted hemisphere.

### 2.6. Neurological Deficits

The neurological function deficits were determined by the 5-scoring system [32] before surgery, 2 h after stroke, and 24 h after reperfusion. The higher scores indicate more serious neurological defects.

### 2.7. ROS Production Measurement by Flow Cytometry Assay

As described previously by us [35], the adult brain dissociation kit (130-107-677, Miltenyi Biotec, Bergisch Gladbach, Germany) was used for brain cell isolation. Rats were sacrificed at 6 and 24 h after reperfusion, and the right hemispheres were cut into small pieces, ground, and filtered through a 70 *μ*m cell strainer (Miltenyi Biotec, Bergisch Gladbach, Germany) to obtain single-cell suspensions. Fluorescent-labeled antibodies were added to the cells and incubated at 37°C for 60 minutes according to the instructions. Cells were then washed and analyzed on a FACSCalibur flow cytometer with CellQuest software (BD, San Jose, CA, USA). Results were expressed by the fluorescence value.

### 2.8. Expression of NOX Subunits and Glucose Transporter Subtypes

After homogenization of the isolated cerebral microvessels, TRIzol reagent (Invitrogen, Carlsbad, CA) was used to extract mRNA according to the instructions. Total RNA was then converted into cDNA by a High-Capacity cDNA Reverse Transcription Kit (Applied Biosystems, Foster City, CA). Using cDNA, gene expression was quantified by Prism 7500 real-time PCR (Applied Biosystems, CA, USA). All reactions were performed under the following conditions: 95°C for 15 minutes, 40 cycles of 95°C for 10 seconds, and 60°C for 30 seconds. The sequences for the primers of rat NOX subunits (gp91^phox^, p67^phox^, p47^phox^, and p22^phox^), glucose transporter subtypes (GLUT1 and GLUT3), and GADPH are shown in Table 1. GADPH was used as the control gene to determine the relative expression of mRNA.

### 2.9. Statistical Analyses

All data are expressed as mean ± SE. All of the analyses were performed using GraphPad Prism v7.0 (GraphPad Software, San Diego, CA). The differences between the groups were assessed using one-way analysis of variance (ANOVA) with the significance level set at *P* < 0.05. Post hoc comparisons between groups were further performed using the least significant difference method.

## 3. Results

### 3.1. Physiological Parameters

There were no significant differences in blood pH, pO_2_, and pCO_2_ between the groups (data not shown).

### 3.2. Body Temperature

In the interischemia hypothermia group, a 2.23°C drop was seen in the temperature within 30 minutes of drug administration at 1 h after stroke (1 h before reperfusion) and continued to fall to the lowest of 3.68°C below the initial temperature at 1.5 h reperfusion (Figure 1). Meanwhile, in the inter-reperfusion hypothermia group, a 2.92°C drop was seen within 30 minutes of pharmacological hypothermia induction at 30 min reperfusion and continued to fall to the lowest of 3.75°C under the initial temperature at 2 h reperfusion. For both groups, the body temperatures remained under the initial measurements for up to 12 h reperfusion. Notably, hypothermia was achieved 1 h earlier in the interischemia group compared to the inter-reperfusion hypothermia group.

### 3.3. Cerebral Infarct Volume and Neurological Deficits

**Figure 2:**
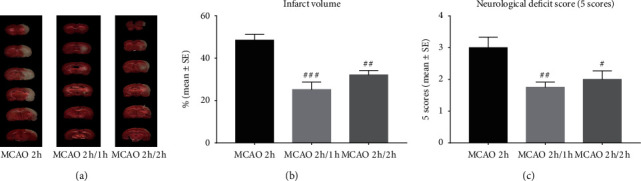
TTC histology demonstrated cerebral infarct volume after stroke with or without hypothermia (a, b). Neurological deficits after stroke and pharmacological hypothermia, using the 5-score system (c). ^#^*p* < 0.05, ^##^*p* < 0.01, and ^###^*p* < 0.001 as compared to the MCAO 2 h group (*n* = 8).

As compared to the stroke group without hypothermia, with the largest cerebral infarct volume at 24 h reperfusion (48.5%) (Figures 2(a) and 2(b)), both hypothermia groups had significantly decreased infarct volumes. The interischemia hypothermia group had a greater decrease in infarct volume of 25.2% (^###^*p* < 0.001) vs. 32.1% (^##^*p* < 0.01) in the inter-reperfusion hypothermia group. As compared to the stroke group at 24 h reperfusion (3.0) (Figure 2(c)), again, both interischemia hypothermia group (^##^*p* < 0.01) and inter-reperfusion hypothermia group (^#^*p* < 0.05) had reduced neurological deficit scores, with interischemia hypothermia being more neuroprotective.

### 3.4. Expression of NOX Subunits and Glucose Transporter Subtypes

**Figure 3:**
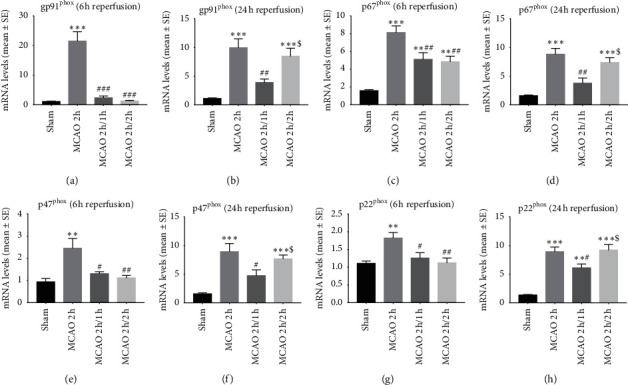
The mRNA expressions of NOX subunits (gp91^phox^, p67^phox^, p47^phox^, and p22^phox^) determined by real-time PCR at 6 and 24 h reperfusion. ^*∗*^*P* < 0.05, ^*∗∗*^*P* < 0.01, and ^*∗∗∗*^*P* < 0.001 as compared to the sham group; ^#^*p* < 0.05, ^##^*p* < 0.01, and ^###^*p* < 0.001 as compared to the MCAO 2 h group; ^$^*P* < 0 .05 as compared to the MCAO 2 h/1 h group (*n* = 8).

The stroke group without hypothermia had a significant increase in the mRNA expression of NOX subunits (gp91^phox^, p67^phox^, p47^phox^, and p22^phox^) at 6 and 24 h reperfusion (Figure 3). Compared to this group, both hypothermia groups observed significantly reduced mRNA expressions of NOX subunits at 6 and 24 h reperfusion. At 24 h reperfusion (Figures 3(b), 3(d), 3(f), and 3(h)), the interischemia hypothermia group had a significant additional reduction in NOX subunit mRNA expression.

**Figure 4:**
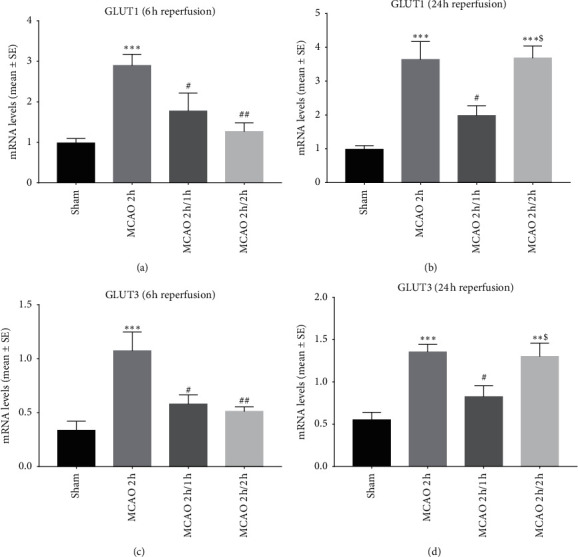
The mRNA expressions of glucose transporter subtypes (GLUT1 and GLUT3) determined by real-time PCR at 6 and 24 h reperfusion. ^*∗∗*^*P* < 0.01 and ^*∗∗∗*^*P* < 0.001 as compared to the sham group; ^#^*p* < 0.05 and ^##^*p* < 0.01 as compared to the MCAO 2 h group; ^$^*P* < 0 .05 as compared to the MCAO 2 h/1 h group (*n* = 8).

A significant increase in the mRNA expression of glucose transporter subtypes (GLUT1 and GLUT3) was seen in ischemic rats with 6 and 24 h reperfusion (Figure 4). Both hypothermia groups observed significantly reduced mRNA expressions. Although there was no difference between interischemia and inter-reperfusion in the reduction of glucose transporter subtype mRNA at 6 h reperfusion (Figures 4(a) and 4(c)), at 24 h reperfusion (Figures 4(b) and 4(d)), interischemia hypothermia induced a significantly greater reduction of mRNA expression.

### 3.5. ROS Production

**Figure 5:**
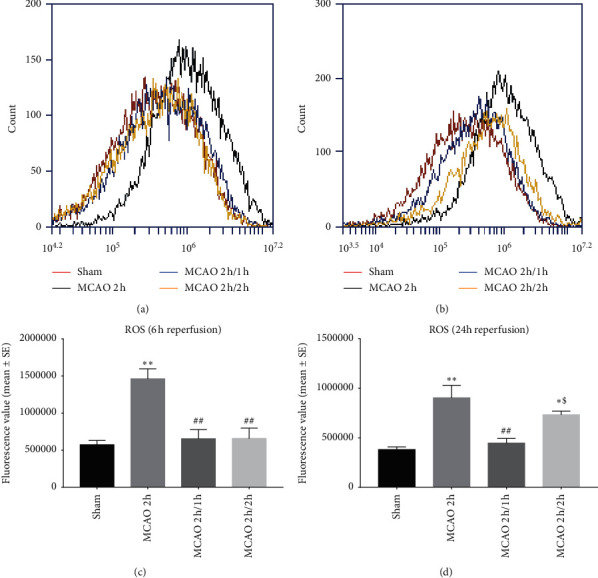
ROS production by flow cytometry assay. ^*∗*^*P* < 0.05 and ^*∗∗*^*P* < 0.01 as compared to the sham group; ^#^*p* < 0.05 and ^##^*p* < 0.01 as compared to the MCAO 2 h group; ^$^*P* < 0 .05 as compared to the MCAO 2 h/1 h group (*n* = 8). The *x*-axis is the fluorescence value. Red: sham; black: MCAO 2 h; blue: MCAO 2 h/1 h; yellow: MCAO 2 h/2 h.

Stroke induced a significant increase in ROS production at 6 and 24 h reperfusion (^*∗∗*^*P* < 0.01) (Figure 5). The two hypothermia protocols significantly decreased the ROS production at 6 h reperfusion (Figures 5(a) and 5(c)) (^##^*p* < 0.01), while the interischemia hypothermia group enhanced the ROS reduction (^$^*P* < 0 .05) at 24 h reperfusion (Figures 5(b) and 5(d)).

## 4. Discussion

In this study, we reported that hypothermia conducted interischemia or inter-reperfusion reduced cerebral infarction volume, neurological deficits, ROS production, and mRNA expression of NOX subunits as well as glucose transporter subtypes. In addition, we found that interischemia hypothermia had further reduced the indications for brain damage compared to inter-reperfusion hypothermia at 24 h after stroke. These findings support our hypothesis that interischemia hypothermia induced by the pharmacological approach provided stronger neuroprotection of the ischemic brain.

Hypothermia therapy depends largely on several factors, such as the time of initiation, the duration, and the depth of hypothermia [11, 36]. Studies have shown that hypothermia should be initiated as soon as possible, target temperature reached quickly, and the lower temperature maintained for a considerable period for better neuroprotective effect [20]. Some earlier studies have shown that reducing the body temperature by only a few degrees, if it is induced in the early ischemic period, can have significant neuroprotective effects [37–39].

The benefits of interischemia hypothermia have been shown in cardiac studies [40, 41]. Studies have reported that hypothermia before reperfusion can reduce the size of myocardial infarction, save more dying myocardial cells, and improve the outcome of myocardial infarction. Many studies of animal models and human trials have shown that interischemia hypothermia is not only safe and feasible but also has good protective effects on ischemic myocardium [42–45]. Compared to postreperfusion hypothermia, interischemia hypothermia has been shown to be more beneficial to cardiomyocytes, which may be related to decreased core infarction volume, oxidative damage, and cell damage after reperfusion. To further illustrate, a previous study showed that the induction of hypothermia 25 minutes after ischemia onset with 40 minutes total ischemia time resulted in a 39% reduction in myocardial infarction area in pigs, whereas hypothermia after reperfusion did not reduce myocardial infarct volume [43]. Furthermore, reaching target temperature before reperfusion is of crucial importance in reducing the infarct size in the treatment of ST-segment elevation myocardial infarction (STEMI) patients [46].

In cerebral ischemia, some evidence supports that early prophylactic mild-to-moderate hypothermia induced shortly after injury in patients with severe traumatic brain injury could decrease mortality and improve neurologic recovery [47]. Ding et al. showed that local cerebral hypothermia induced by infusion of cold saline prior to reperfusion that is maintained for 10 minutes and followed by complete reperfusion can reduce brain injury, improve neurological function, and maintain long-term functional recovery [48]. The advantage of interischemia hypothermia is that the target temperature is reached prior to reperfusion, which not only reduces ischemia injury but also reperfusion injury. Therefore, it is more beneficial to start hypothermia treatment as soon as possible after brain injury.

At present, hypothermia therapy in clinical practice is mainly systemic physical cooling or endovascular intracarotid infusion of cold saline [49–52]. It is difficult to establish, slow in cooling, cannot reach the target temperature quickly, and can easily lead to serious complications such as arrhythmias and pulmonary infections [53–55]. Therefore, current clinical application of hypothermia is limited. In contrast, drug hypothermia is more convenient and can achieve the target temperature before vascular recanalization without extensive medical and nursing efforts. This may have a certain clinical application prospect, where it could become a better choice for hypothermia induction before vascular recanalization [11]. However, drug hypothermia also has some limitations. The efficiency of single-drug application is low with many complications. When drugs are combined in low doses, they can build a synergistic effect, improving the efficiency of low temperature, reducing the side effects of any single drug, and minimizing complications. Our previous study demonstrated that chlorpromazine and promethazine (C + P) significantly reduced the volume of cerebral infarction in rats and attenuated neurological deficit [31, 56]. Dihydrocapsaicin (DHC), a potential capsaicin channel transient receptor agonist (TRPV1), is also currently being investigated as a promising drug cryogenic inducer [57, 58]. Studies have shown that DHC in high doses can independently achieve effective hypothermia therapy, although its applications alone are limited due to the significant toxicity and complications [11, 33]. However, combined with low doses of C + P, it might play a synergistic effect, improve the efficiency of hypothermia, reduce the side effects of the single drug, and reduce the complications [59]. Therefore, in this study, we chose C + P combined with DHC to induce pharmacological hypothermia.

The full mechanism of hypothermia's neuroprotective effects is still being explored and described. A review has shown that hypothermia may act on several pathways in the ischemic cascade and have different effects on the inflammatory response at different time points [60]. ROS plays an important role in the pathophysiological process of ischemic neuron injury [23]. NADPH oxidase complex produces superoxide (O_2_) and is involved in ROS production during ischemia and reperfusion [61]. Explosive ROS production has been shown to occur mainly in the first 10–15 minutes of reperfusion in the MCAO rat model [22]. In this model, our study shows that interischemia hypothermia and inter-reperfusion hypothermia induced by both low doses of phenothiazine drugs and DHC reduced ischemia-reperfusion injury, protected brain tissue, and induced neuroprotection compared to stroke without treatment. Interischemia hypothermia treatment may be better able to induce stronger neuroprotection after stroke.

## 5. Conclusion

In conclusion, both interischemia and inter-reperfusion pharmacological hypothermia treatments play a role in neuroprotection after stroke. Interischemia hypothermia treatment may be better able to induce stronger neuroprotection after ischemic stroke. In the current clinical environment, delaying reperfusion therapy to wait for hypothermia induction is not feasible. Fortunately, pharmacological hypothermia with low dose is quick and easy to use and has few side effects when used in combination. Patients could be cooled upon arrival to the emergency room or even prior to, perhaps in the ambulance by Emergency Medical Services (EMS), en route to the primary or advanced stroke unit, before thrombolysis or thrombectomy. This study provides a referential and effective treatment strategy for cerebral protection before vascular recanalization in acute ischemic stroke, as well as the basis for the realization of clinical transformation.

## Figures and Tables

**Table 1:** Primers for real-time polymerase chain reaction (PCR) analysis.

Genes	Forward primer (5'-3')	Reverse primer (5'-3')
gp91^phox^	TGACTCGGTTGGCTGGCATC	CGCAAAGGTACAGGAACATGGG
p67^phox^	AGCAGAAGAGCAGTTAGCATTGG	TGCTTTCCATGGCCTTGTC
p47^phox^	TCACCGAGATCTACGAGTTC	ATCCCATGAGGCTGTTGAAGT
p22^phox^	TGTTGCAGGAGTGCTCATCTGTCT	AGGACAGCCCGGACGTAGTAATTT
GLUT1	CAGAGCGACAAGACACCTGA	ACTGAAGAAAGGTGCCCAGG
GLUT3	GTGGAGCGGTGAAGATCAGATA	GGCAACAGTAACAGCGAACA
GADPH	CAAGAAGGTGGTGAAGCAG	AAAGGTGGAAGAATGGGAG

## Data Availability

The original data used to support the findings of this study are included within the article.
